# Where’s wallaby? Using public records and media reports to describe the status of red‐necked wallabies in Britain

**DOI:** 10.1002/ece3.6877

**Published:** 2020-11-02

**Authors:** Holly M. English, Anthony Caravaggi

**Affiliations:** ^1^ Laboratory of Wildlife Ecology and Behaviour School of Biology and Environmental Science University College Dublin Dublin Ireland; ^2^ School of Applied Sciences University of South Wales Pontypridd UK

**Keywords:** biological records, macropod, non‐native species, *Notamacropus rufogriseus*, population dynamics

## Abstract

Investigating the range and population dynamics of introduced species provides insight into species behavior, habitat preferences, and potential of becoming established. Here, we show the current population status of the red‐necked wallaby (*Notamacropus rufogriseus*) in Britain based on records from an eleven‐year period (2008–2018). Records were obtained from Local Environmental Records Centres (LERCs), the National Biodiversity Network (NBN), and popular media. All records were mapped and compared to a historical distribution map (1940–2007), derived from published data. A total of 95 confirmed wallaby sightings were recorded between 2008 and 2018, of which 64 came from media sources, 18 from Local Environmental Records Centres (LERCs), seven from the National Biodiversity Network (NBN), and six from the published literature (Yalden, Br. Wildl., 24, 2013, 169). The greatest density of wallaby sightings was in southern England, with the Chiltern Hills Area of Outstanding Natural Beauty a particular hot spot (*n* = 11). More sightings were recorded in August than in any other month. Much of the species’ ecology and responses to British biota and anthropogenic pressures are unknown, and therefore, further research is warranted. The methods used here are widely applicable to other non‐native species, particularly those that the public are more likely to report and could be an important supplement to existing studies of conservation and management relevance.

## Graphical abstract

We present the current population status of the non‐native red‐necked wallaby (*Notamacropus rufogriseus*) in Britain based on records from an eleven‐year period. A total of 95 confirmed wallaby sightings were recorded between 2008 and 2018, of which 64 came from media sources, 18 from Local Environmental Records Centres, seven from the National Biodiversity Network, and six from published literature. The methods used here are widely applicable to other species, particularly those that the public are more likely to report, and could be an important tool for studies of non‐native and invasive species with conservation and management relevance.

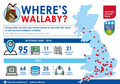

## 1 INTRODUCTION

The red‐necked wallaby (*Notamacropus rufogriseus*) is a medium‐sized mammal native to southeast Australia, including Tasmania, King Island, and the Furneaux Group (McKenzie et al., 2016). Introduced populations have been reported in Britain, Ireland, France, and New Zealand (Genovesi et al., 2009). The diet of wallabies in Britain (Weir et al., 1995) and the Isle of Man (Havlin et al., 2017) is comparable to that in their native range (Jarman & Phillips, 1989; Sprent & McArthur, 2002), comprising grasses, rushes, sedges, forbs, and other vegetation. Wallabies are typically solitary, although fluid social groups also occur. In their native range, the stability of these social groups is affected by seasonality, with less social cohesion in winter months when individuals move greater distances to forage (Johnson, 1989). It is unclear whether introduced wallabies exhibit the same behavior, as macropodid marsupials isolated on islands can exhibit pronounced behavioral changes (Blumstein & Daniel, 2005). Previous camera trap studies of captive but free‐ranging wallabies in Whipsnade Zoo (Rowcliffe et al., 2008) and a wild population on the Isle of Man (Havlin et al., 2017) did not detect cohesive social groups. It is not clear, however, given the need for additional considerations when inferring behavior from camera trap data (Caravaggi et al., 2020), whether those findings represent accurate depictions of intragroup behavior in these non‐native populations.

The red‐necked wallaby was introduced to Britain as a charismatic species for display in zoos and private collections (Baker, 1990). Wallabies were introduced to the collections at Whipsnade Zoo in 1931, although were present in the Channel Isles and Woburn Park, Bedfordshire prior to this. A breeding population was maintained in Whipsnade with wallabies periodically sold to private collectors (Yalden, 2013). Many wallabies succeeded in escaping captivity, usually temporarily, and often due to poor fence maintenance, particularly during World War II (Yalden & Hosey, 1971). The first range map for red‐necked wallabies in Britain showed two populations, one in the Peak District and one in Sussex (Arnold, 1984). The Peak District wallabies, originating from five individuals intentionally released from a private collection in 1939–1940, were the most well‐known and well‐studied wallaby population in Britain (Yalden, 1988, 1990; Yalden & Hosey, 1971). This population appeared to die out naturally, though occasional sightings are still reported (AC *pers. obs*.). Yalden (2013) suggested that sightings after 2008 were likely to be of illegally released, captive wallabies by locals hoping to restore a population, rather than survivors from the gradual die‐off which occurred between 1996 and 2008. In Scotland, four wallabies were deliberately released on an island in Loch Lomond in 1975. The population numbered at least 26 individuals in 1992 (Weir et al., 1995) and is still extant. The success of this population may be due in part to their insular home and the maritime climate, which is consistent with the success of wallabies on Lambay Island, Ireland, and on the Isle of Man (Connolly, 2014; Havlin et al., 2017; Weir et al., 1995). There have been no reports of live wallaby sightings in Wales.

Since the decline and eventual extinction of the Peak District population, the extent to which wallabies have persisted in Britain is uncertain. Their distribution may have been limited by several factors, such as predation by dogs (*Canis lupus familiaris*) and foxes (*Vulpes vulpes*), a harsh winter climate, and road fatalities (Havlin et al., 2017; Yalden, 2010). However, these factors are also present on the Isle of Man, where wallabies have proliferated (Havlin et al., 2017), as well as in their native range (with the potential exception of the harsh winter climate; Cox et al., 2015; Jarman et al., 1987; Meek & Wishart, 2017; Ramp, 2010; Robertshaw & Harden, 1985). At least partial loss of antipredator behavior is common in introduced, insular marsupials (Blumstein & Daniel, 2005; Blumstein et al., 2000, 2002), indicating a potential trade‐off between investment in other life‐history functions and increased predation risk. Further, the possible impact of harsh winters on wallaby populations suggests that future climate change may be a potentially significant factor in determining the establishment or extinction of wallabies in Britain, as warmer, wetter winters could potentially benefit the species. Landscape composition and use is also a significant factor affecting establishment potential. Red‐necked wallabies typically inhabit scrub and sclerophyll forest in their native range. Southern Britain has been intensively farmed and is now largely comprised of a woodland‐agricultural matrix (Neumann et al., 2016) that could, in principle, support a population of wild wallabies.

Non‐native species are widely considered one of the greatest contributing factors to modern biodiversity loss (Barrett et al., 2018; Mack et al., 2000). However, relatively few non‐native species become successfully established in new regions and it is difficult to predict how a non‐native species will affect or be affected by receiving ecosystems. There is potential for numerous and diverse negative effects via direct interactions with native biota, for example, through competition, predation, or disease transmission (Caravaggi et al., 2018; Le Louarn et al., 2016; McCreless et al., 2016; Vilcinskas, 2015; Welch & Leppanen, 2017). Non‐native species do not always exhibit detectable negative effects on receiving ecosystems and/or native species, however. Indeed, few of the species introduced to mainland Britain have had apparent detrimental impacts and several, such as the European Rabbit (*Oryctolagus cuniculus*) and Little Owl (*Athene noctua*), are now considered as part of Britain's biodiversity (Davis, 2009; Keller et al., 2007; Manchester & Bullock, 2000; Schlaepfer et al., 2011). Furthermore, non‐native species do not necessarily affect an ecosystem in a uniform way; they may have deleterious, neutral, and beneficial impacts on different species within and across an ecosystem (Bonanno, 2016; Zavaleta et al., 2001). Quantifying the distribution and temporal population dynamics of non‐native species is important in furthering our understanding of ecological communities and the potential for negative impacts on native species. Such information can inform conservation management and policy (Shackelford et al., 2013), particularly under different land management regimes and climate change (Keith et al., 2008).

The Internet, social media in particular, and wildlife records submitted by the general public are becoming increasingly useful resources to ecologists, allowing rapid data collection on focal species of interest. For example, Google Images has been used to assess phenotypic variation and diet in species across their range (Leighton et al., 2016; Naude et al., 2019), and image mining has been used to assess the distribution of non‐native freshwater turtles (Allain, 2019). Such methods allow data to be recorded across larger spatial and temporal scales than are often practical or affordable for individual field studies (Naude et al., 2019) including entire countries, particularly where citizen scientists become involved. Charismatic alien species are noteworthy to the general public and media and, as such, sightings are often reported in local newspapers or various online web pages, particularly social media websites. Furthermore, record center databases tend to be biased toward charismatic and novel species, with records frequently outnumbering those of more common, familiar species (Isaac & Pocock, 2015).

The current status of the red‐necked wallaby in the UK and its potential impacts on native biota are unknown. Addressing this knowledge gap is the first step in quantifying population parameters, assessing the likelihood of establishment (if, indeed, the species is not already established), and developing appropriate conservation/management processes and policies. Here, we describe the current distribution of wallabies across Britain, compiled from records collated through local and national databases, media reports, and social media. These sources were used due to the dearth of information available on British wallabies in the scientific literature, particularly outside the Peak District. We discuss our findings in the context of potential impacts on native British biota and the potential for a population of wallabies to become (re)established in Britain.

## 2 MATERIAL AND METHODS

**Figure 1:**
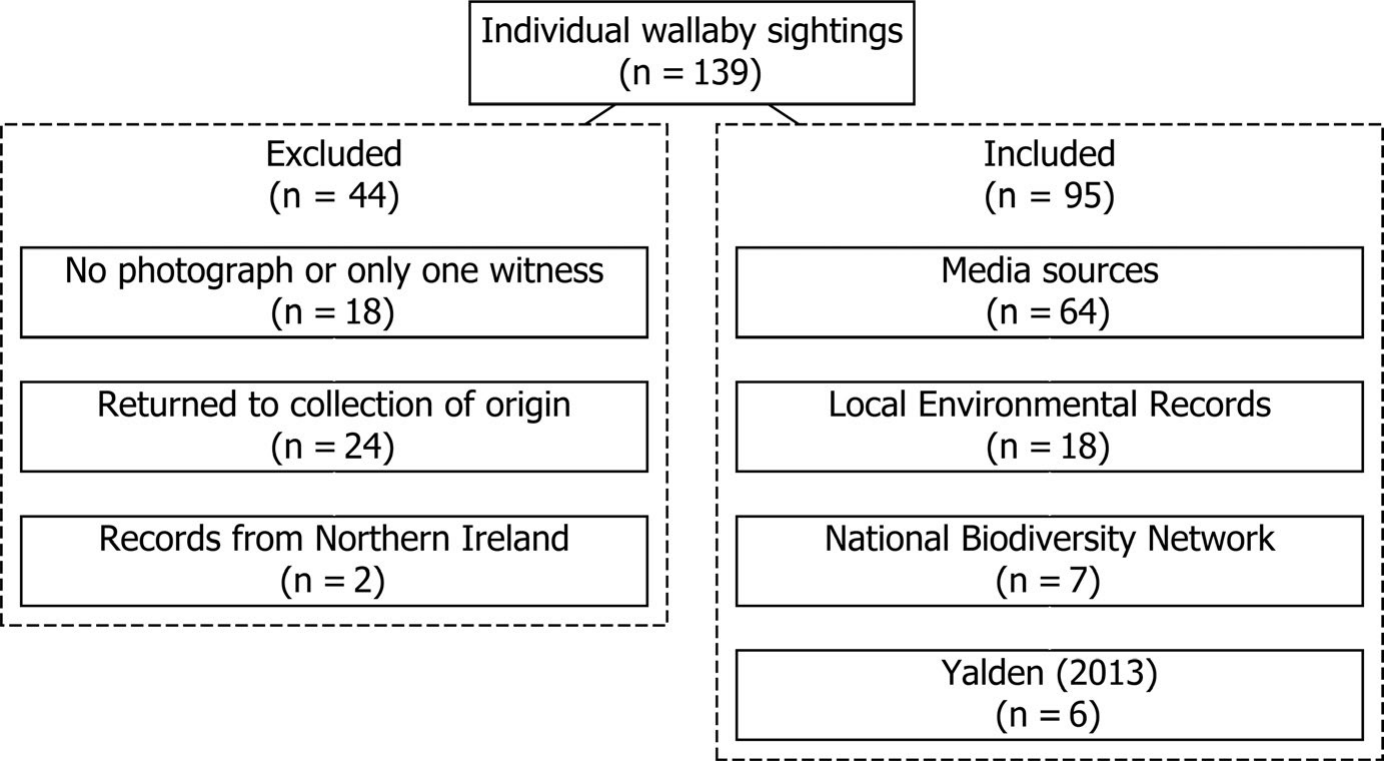
A flowchart depicting the decision process for inclusion or exclusion of individual records for the modern data set

Our review was based on the question “What is the current status of the free‐ranging wallaby population in Britain?” To answer this question, we performed a comprehensive search of electronic media websites (e.g., Google News, BBC News, Sky News, newspaper websites, local news websites, local interest group websites, Facebook, Reddit, Twitter) from 1 January 2008 to the 31 December 2018. We searched for any mention of wallabies in Britain using the keywords: ‘wallab*’; ‘red‐necked wallab*’; ‘macropod’; *‘Macropus’*; and ‘*Notamacropus*’, combined with: ‘UK’; ‘GB’; ‘Britain’; ‘Wales’; ‘England’; ‘Scotland’; ‘Northern Ireland’; ‘escape’; ‘non‐native’; and combinations and variations, thereof. An initial Google search returned > 6,000,000 results due to the inclusion of results featuring the Australian men's rugby team, known as “The Wallabies.” Thus, subsequent searches excluded the keyword “rugby.” News items were considered reliable and subsequently retained if there was photographic evidence and/or there were multiple eyewitness reports of the same animal. Reports of escaped wallabies that were returned to their collections of origin were discounted; escapees that had not been returned to the collection of origin (as determined by the presence or absence of subsequent, related articles) were retained (Figure 1). Duplicate data between source data sets were identified by a combination of date and location and were also removed. We also solicited the submission of verifiable records (i.e., those that could be accompanied by a photograph or where there were multiple observers) via a bespoke website, Facebook, and Twitter. From retained records, we collected all relevant information, including the date (i.e., month, year) of the item/observation; the source (e.g., the name of the newspaper); the state of the animal(s) (i.e., alive or dead; both states were treated equally); the number of animals observed; location; and any additional notes made by observers. Observations were classed as independent based on time, day, location, and inferential analysis of images. Data were also obtained from Local Environmental Records Centres (LERCs; http://www.alerc.org.uk/) and the National Biodiversity Network (NBN; http://www.nbn.org.uk/) for the given period. LERC and NBN data are subject to internal screening and verification by expert volunteers. While the authors were not able to verify the accuracy of these observations themselves and it is possible that the data contained misidentifications, these represent the best available broadscale data on mammals in the UK.

For our historical dataset, we examined a total of 60 recorded locations where wallabies had been reported between 1890 and 2013 (Yalden, 2013). Some locations corresponded to single escapees while others represented populations through time, with start and end dates of presence recorded. We retained 41 of these locations to compose the historical data set. Two discounted records were from the Channel Isles and the Isle of Man (see Havlin et al., 2017) which we do not consider here. Seventeen records from Yalden (2013) occurred in 2008 or later, that is, during our modern distribution period. Eleven of the records from the historical data set matched those obtained via media reports or NBN/LERC providers. The other six records from Yalden (2013) occurred between 2008 and 2013 but had not been accounted for in our other sources, so were incorporated into our modern data set.

We compiled a map of wallaby sightings for mainland Britain over an eleven‐year period (2008–2018) and compared this to historical data, between 1890 and 2007 (Yalden, 2013). The relative density of occurrences within contemporary data was calculated using kernel density estimators with probability thresholds set at 25%, 50%, and 75% of all records. Grid references from the historic data set and LERC records were converted to decimal latitude and longitude values using https://gridreferencefinder.com/. Location data from media reports were almost always vague, usually describing a town or village or a road between two locations. Grid references used to map records were derived from locality and any additional information in the source that supported location identification. While locations were mapped as precisely as possible, it is impossible to quantify any (in)accuracies, and therefore, our maps should be considered approximations at the local scale. Data and R code are available at https://doi.org/10.5281/zenodo.4034730.

## 3 RESULTS

**Figure 2:**
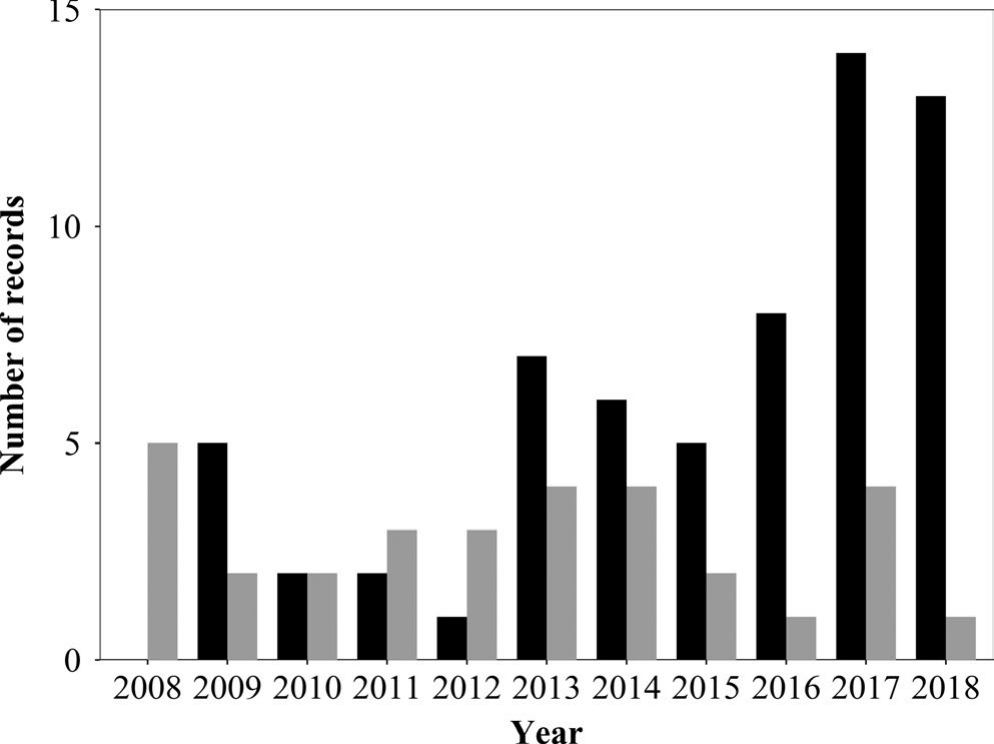
Total number of red‐necked wallaby records in Britain, per annum, between 2008 and 2018. Media‐sourced records are shown in black and local and national database (i.e., LERC/NBN) records are shown in gray

A total of 139 individual wallaby sightings were recorded between 2008 and 2018 (Figure 1). Eighteen records were discounted as there was no photographic evidence and/or there was only one witness. Twenty‐four records were discounted as the animals in question were noted as having been returned to the collection of origin. Finally, two records from Northern Ireland were discounted. Of the 95 sightings included here, 64 came from media sources (including seven from social media reports and one record from YouTube), 18 from Local Environmental Records Centres (LERCs), seven from the National Biodiversity Network (NBN, Figures 1 and 2), and six from Yalden (2013, see Section 2). Almost all records were of individual wallabies, with the exception of two records of females with young in the pouch in May 2009 and June 2010, respectively (with an adult male also reported in 2010), possibly indicating breeding in the wild.

**Figure 3:**
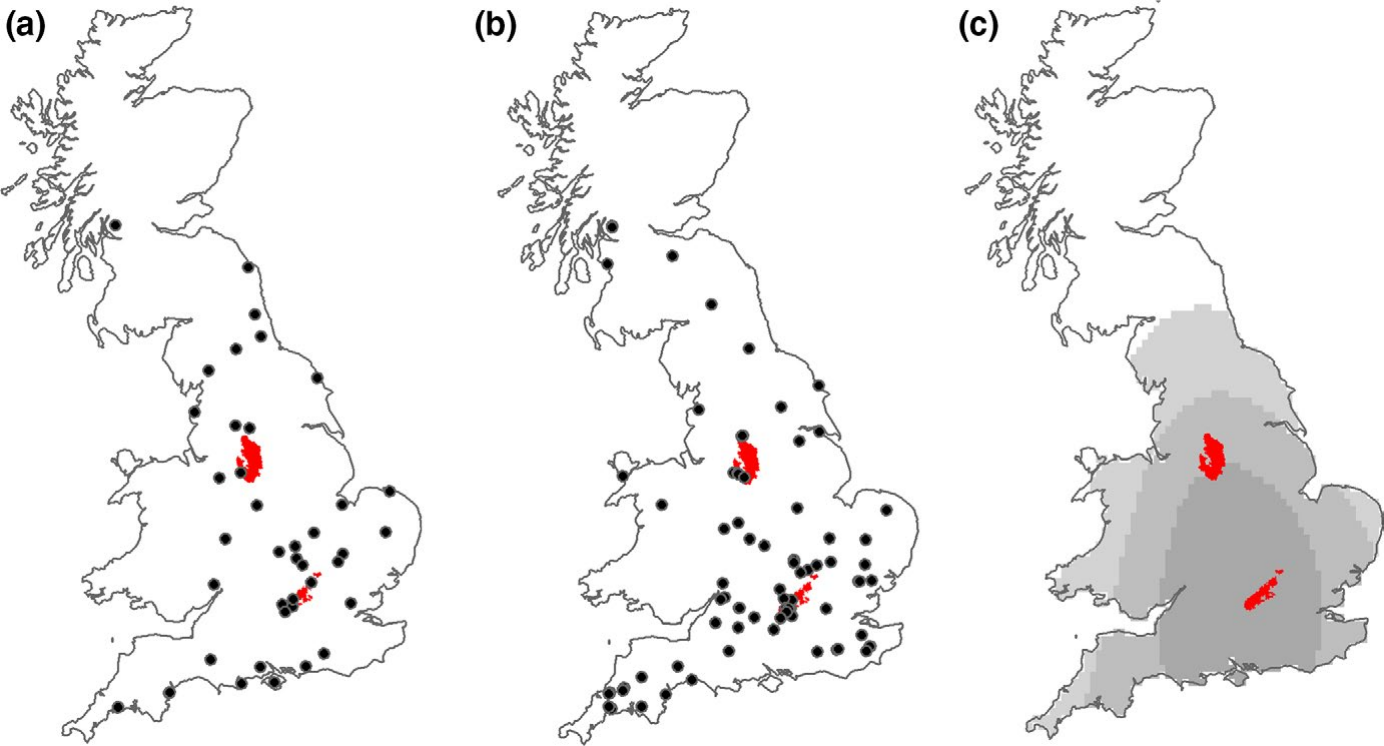
(a) Historical (i.e., pre‐2008, *n* = 41) red‐necked wallaby sightings in Great Britain; (b) contemporary distribution (2008–2018, *n* = 95); and (c) Kernel density estimation polygons describing the probability of wallaby occurrence, based on contemporary wallaby sightings, where light gray = 75% probability, gray = 50% probability, and dark gray = 25% probability. The Peak District National Park (North) and Chilterns Area of Outstanding Natural Beauty (South) are indicated by red polygons

**Figure 4:**
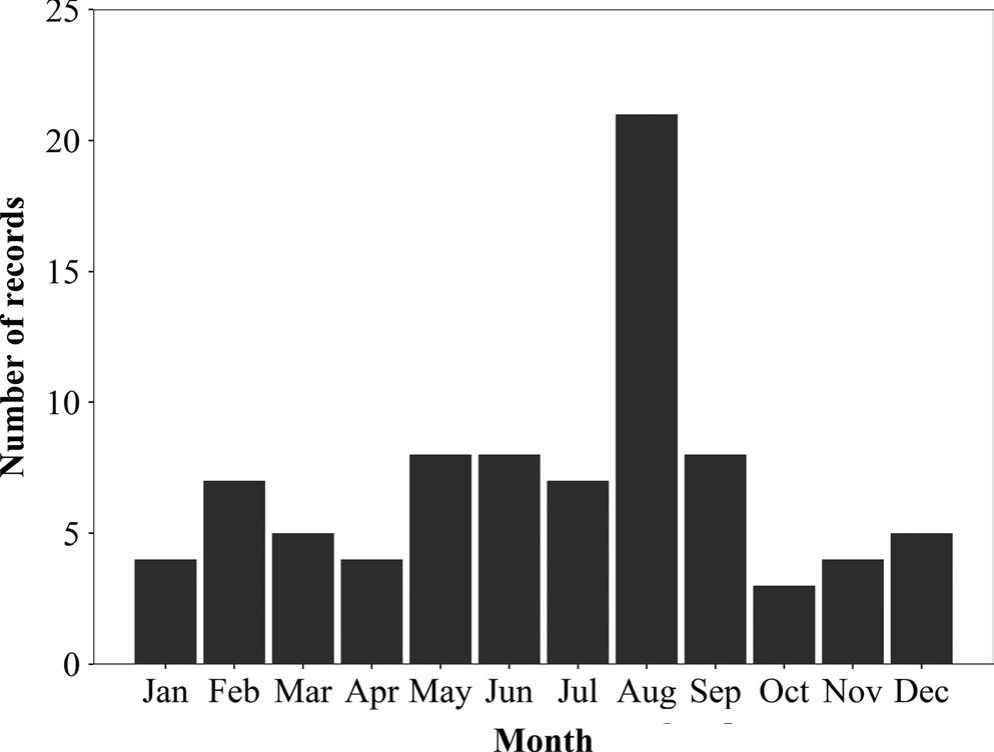
Wallaby sightings by calendar month across Great Britain between 2008 and 2018 for those records where the specific month was recorded (*n* = 84)

Kernel density estimators showed that the greatest density of wallaby sightings (75% probability) was found in southern England (Figure 3c). This was due to 11 sightings across an area of approximately 340 km^2^ in the Chiltern Hills Area of Outstanding Natural Beauty (AONB) between 2013 and 2017. The month in which records were reported was not available for the historical data set, or the six modern records that were obtained from the same source (Yalden, 2013). The month in which a sighting occurred was available for 84 modern records, of which 25% (*n* = 21) occurred in August. Counts in the remaining calendar months varied between 3 and 8 sightings over the same eleven‐year period (Figure 4).

## 4 DISCUSSION

Red‐necked wallabies currently exhibit a more southerly distribution in Great Britain than was previously the case, as described by historic data, with particular hot spots around the Chiltern Hills AONB. The species was once found in the Peak District and in good numbers (Yalden, 1988, 1990; Yalden & Hosey, 1971). However, the dearth of modern data adds weight to the suggestion that this population is now extinct (Yalden, 2013). Distributions of non‐native species and their changes over time indicate whether populations are static, spreading, or retracting. Our maps indicate the potential spread of feral wallabies in Britain. However, this may be an effect of increased record availability when using online media sources or increased public interest over time. An increasing proportion of records were derived from media sources in recent years, highlighting the limitations of relying on centralized databases (e.g., NBN, LERCs), alone. Moreover, the disagreements between LERC and NBN data suggest a disjunction in the process of centralizing biodiversity data in Britain that could have important implications for future monitoring studies.

More wallaby sightings were recorded in summer than winter, with the majority of records occurring in August (Figure 4). The reasons for this are unclear. Detection probabilities may be higher in summer months due to longer daylight hours and human observers spending more time outside. However, this does not explain the difference in the number of sightings recorded in August compared to other summer months. In their native range, wallabies have been observed to maintain small home ranges which remain relatively stable across seasons and years (Johnson, 1987), although males have significantly larger home ranges than females (Johnson, 1987; Le Mar et al., 2003) and females have been reported with larger home ranges in winter than summer for reasons related to food availability (Johnson, 1987). Home range size is thought to vary with the presence of other herbivores in the native range of the related yellow‐footed rock wallaby (*Petrogale xanthopus*), with home range size decreasing after sheep removal and goat control (Hayward et al., 2011). However, introduced wallaby spatial ecology and/or habitat selection could vary throughout the year and does not necessarily mirror that of the species in its home range. Deterministic factors such as differences in ecosystem composition (Barrios‐Garcia & Ballari, 2012), the presence or absence of competitors (Skalova et al., 2013) and predators (Pintor & Byers, 2015), climate (Bertolino, 2009), and human activity (Manchester & Bullock, 2000) can result in unpredictable responses by naive organisms in a novel environment.

Certain areas, particularly Wiltshire, Cornwall, and the Chiltern Hills AONB, represented concentrated zones of sightings that may indicate areas where wallabies have become established or are in the process of establishing a permanent population. Certainly, the size of the area in the Chiltern Hills where 11 sightings were recorded between 2013 and 2017 (340 km^2^) greatly exceeds the home range of the species in its natural environment (e.g., 0.6 km^2^ ± 0.1 km^2^, Le Mar et al., 2003), hence at least some of these sightings can be reasonably attributed to multiple individuals. The wallaby sightings recorded in Cornwall in the summer of 2017 were reported in the media as representing one individual despite the absence of identifying markings and the fact that some of these sightings occurred 28 miles (45 km) apart. The maximum dispersal distance reported by Suckling (1982) in a plantation in the species’ native range was 1.9 km. Males have been shown to have larger dispersal distances when released in areas with no females present in at least one marsupial species (the burrowing bettong, Short & Turner, 2000). However, even if this behavior held true for red‐necked wallabies, it is unlikely to account for movement across a linear distance of 45 km. Elsewhere in Britain, the historic Peak District population was said to be easily located as individuals were typically found close to where they were last sighted (Yalden, 2013). Given the nature of our data and the lack of recent empirical studies of wallabies in Britain, it is impossible to ascertain whether these areas are indicative of population establishment, escapees surviving in the wild but not reproducing, or simply localized public interest resulting in recording bias (Isaac & Pocock, 2015; Pocock et al., 2015). Recording bias may be particularly relevant in cases such as that presented here where the species in question is a charismatic, rare, non‐native mammal (Troudet et al., 2017).

LERCs collate and manage biodiversity data for defined geographic regions across the UK, facilitating research by academics and independent consultants. The NBN performs this function on a national scale in the UK. However, the NBN contained fewer records than the combined LERC data and there was no overlap between the two sources. Furthermore, the records presented here from media sources considerably exceed those from biological record centers (Figure 1). These findings are consistent with recent research on freshwater turtles which found online photograph‐verified alien species reports outnumber records from official record centers (Allain, 2019). This suggests that the average member of the public is more inclined to report sightings of charismatic non‐native species to news outlets or on social media than directly to record centers. If local and national news outlets and record centers collaborate, this presents an opportunity to expand the databases available for biodiversity analysis held in record centers through incorporation of photograph‐verified media reports. Alternatively, record centers might attempt to stay abreast of media reports in their area that feature wildlife (though we acknowledge that this may have cost and personnel implications). Certainly, media outlets could be provided with information on submitting biodiversity records to local and national databases for inclusion in media articles reporting rare species and this information could be noted at the foot of relevant articles, thus promoting public participation in biological recording (Bonney et al., 2009).

Media records are inconsistent in their comments on the responses of local authorities and NGOs to reports of wallabies across Britain. For example, multiple media records stated that the Royal Society for the Prevention of Cruelty to Animals (RSPCA) intervened, while others noted that the RSPCA is not interested in healthy wallabies. One media report claimed that the RSPCA estimated the wild wallaby population in the UK to number in the hundreds (https://www.bbc.co.uk/news/uk-england-york-north-yorkshire-29536628), although this statement is not backed up in any official RSPCA documentation or external reports used by the RSPCA (RSPCA Press Office, pers. comm.). While the photographs which accompany media reports can be independently assessed and verified by researchers, this discrepancy suggests that the precautionary principle should be applied and caution is advised when collecting data from media article text.

**Figure 5:**
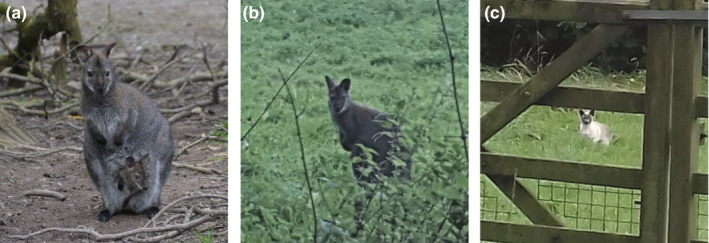
Images of (a) a red‐necked wallaby photographed in a zoological collection (photo by AC), (b) a free‐roaming red‐necked wallaby photographed in Binfield Heath, Oxfordshire (photo courtesy of Matthew Walker), and (c) a misidentified “wallaby”—actually a domestic cat—photographed in the Roaches, Staffordshire (see https://bit.ly/2JP2I9t for misidentification; photo courtesy of Anne Arrowsmith)

Wildlife data submitted by the general public provide a powerful method of data collection on a wider scale than might otherwise be possible via other means (e.g., scat surveys). For example, social media has previously been used to estimate populations of invasive species (Adriaens et al., 2015) including to assess the distribution of wallabies on the Isle of Man (Havlin et al., 2017). However, data from public sightings must always be treated carefully, as often the identification skills of those submitting sightings are unknown. For example, muntjac deer (*Muntiacus reevesi*), another non‐native species in Britain, may occasionally be misreported as wallabies (AC pers. obs.). Other species such as domestic cats (*Felis catus*) and dogs (*Canis lupus familiaris*) have also been incorrectly identified as wallabies online (Figure 5). Here, we addressed this problem by only using sightings with photographic evidence and/or multiple witnesses.

It is not clear whether the establishment of a wild population of wallabies in Britain represents a threat to native biota. For example, hundreds of thousands of red‐necked wallabies may have been present in New Zealand by 1960, following the release of two females and one male to South Island in 1874 (Catt, 1977), and it is thought that their range could expand sevenfold in the next 50 years if intervention strategies are not implemented (Latham et al., 2019). These wallabies compete with farmed sheep and contribute to vegetation loss in agricultural and natural systems (Catt, 1977; Latham et al., 2019). Wallabies also represent a potentially important and novel vector for disease in the British context, including *Toxoplasma gondii* (Basso et al., 2007; Dubey & Crutchley, 2008), leishmaniasis (Montoya et al., 2016; Ramírez et al., 2013), *Giardia* sp. (Thompson et al., 2008), and *Cryptosporidium* sp. (Fayer, 2004). However, wallabies are not listed on the European Commission's list of invasive alien species of Union concern (https://ec.europa.eu/environment/nature/invasivealien/list/index_en.htm). EU member states are obliged to work toward the early detection and rapid eradication of listed species and apply appropriate management actions to limit the impacts of those that have become established. Indeed, free‐ranging populations of red‐necked wallabies occur in Ireland and France (Connolly, 2014; Genovesi et al., 2009) and do not appear to negatively impact local ecosystems. It is unclear why wallabies became invasive in New Zealand, but not in the other regions to which they have been introduced, especially those where invasive muntjac, a species that is ecologically comparable, have thrived. The island susceptibility hypothesis suggests that non‐native species are more likely to establish on islands than continents and, indeed, long‐term wallaby success in Europe seems to be linked to their occurrence on small, maritime islands (Connolly, 2014, Havlin et al., 2017, but see Simberloff, 1995, Jeschke et al., 2012 for criticisms of this hypothesis). It is important to note, however, that the impact potential of wallabies in Britain has not been quantified. Further studies are required to assess the likelihood of interspecific competition between wallabies and native or naturalized British species, the impacts of grazing wallabies on vegetative species of conservation concern and the potential for disease transmission between wallabies and other species, including humans. Scat analysis could be used to assess likelihood of competition with native species and disease transmission potential. Genetic tools such as environmental DNA metabarcoding could also be used to detect wallaby presence in a given location (Browett et al., 2020).

The number of records excluded from the current study demonstrates that wallabies are proficient at escaping from zoos and private collections, and not all escapees are caught. Wallabies are also popular with private collectors, and some members of the public keep breeding populations to sell (pursuant to the Wildlife and Countryside Act 1981) as they are occasionally used for grazing in place of sheep. However, this is not to say that wallabies are not breeding in the wild in Britain. Indeed, sightings of a female wallaby with a juvenile in the pouch were recorded in St. Breward, Cornwall in May 2009 and June 2010, respectively, with an adult male also reported in the 2010 record. Wallaby joeys begin to emerge from the pouch for short periods of time at approximately 230 days of age and leave the pouch permanently at 280 days (Merchant & Calaby, 1981). However, this is not necessarily indicative of breeding in the wild as red‐necked wallabies readily breed in captivity and these individuals may have been recent escapees. Most records do not specify sex, and none specify reproductive state. Further studies are required to quantify important population parameters and establish whether there is a breeding population in Britain.

## 5 CONCLUSION

Red‐necked wallabies appear to be thriving at low‐population levels in Britain, and potential breeding populations exist in southern England. Though common, red‐necked wallabies are poorly studied even in their native range (Le Mar et al., 2003). Furthermore, much of the species’ ecology and responses to British biota (and vice versa) and anthropogenic pressures are unknown; hence, further, focussed research is warranted. Media articles provided more records of this non‐native species than can be found in record centers alone, highlighting key discrepancies and weaknesses in centralized biodiversity recording in Britain. Collaborations between media outlets and record centers could lead to a substantial increase in the number and availability of species occurrence records. This could have important implications for derived management and conservation processes, and policy. The methods used here are widely applicable to other non‐native species, particularly medium‐to‐large, charismatic species that the public are more likely to report.

## CONFLICTS OF INTEREST

We confirm that we have no conflicts of interest to declare. Data were collated from existing sources, and as such, ethical approval was not required.

## AUTHOR CONTRIBUTION

**Holly M. English:** Data curation (lead); Formal analysis (equal); Investigation (equal); Writing‐original draft (lead); Writing‐review & editing (equal). **Anthony Caravaggi:** Conceptualization (lead); Data curation (supporting); Formal analysis (equal); Investigation (equal); Methodology (lead); Project administration (lead); Supervision (lead); Writing‐review & editing (equal).

## Data Availability

All sightings collated from media reports are presented in Table 1. We do not have permission to share records from Local Environmental Records Centres but contact information for data managers can be provided upon request. Data excluding those from LERCs and R code are available at https://doi.org/10.5281/zenodo.4034730 Confirmed wallaby observations in Britain (2008–2018) as reported by online media outlets and social media Records are listed chronologically, by year. Locality = location as reported in the source article. NR = date not reported.
